# Predictive value of early DCE and DSC perfusion MRI parameters for midterm clinical outcomes in lung cancer brain metastases treated with stereotactic radiosurgery

**DOI:** 10.1007/s11060-025-05054-5

**Published:** 2025-05-23

**Authors:** Yunus Emre Senturk, Enes Muhammed Canturk, Ahmet Peker, Sabahattin Yüzkan, Yavuz Samancı, Selçuk Peker

**Affiliations:** 1 Department of Radiology, Koc University Hospital, Davutpaşa Caddesi, No 4, Istanbul, 34010 Turkey; 2 Department of Neurosurgery, Koc University Hospital, Istanbul, Turkey

**Keywords:** Stereotactic radiosurgery, Brain metastasis, Perfusion MRI, Lung carcinoma

## Abstract

**Purpose:**

Stereotactic Radiosurgery (SRS) is an effective way of controlling the brain metastasis (BM) of lung carcinoma. This study evaluates the performance of dynamic contrast-enhanced MRI (DCE-MRI) and dynamic susceptibility contrast MRI (DSC-MRI) parameters to distinguish responders from non-responders at midterm follow-up in lung carcinoma BMs.

**Methods:**

Twenty-six patients (mean age 62 ± 10 years) with 54 lung carcinoma BMs (NSCLC 67%, SCLC 33%) underwent SRS. The DCE-MRI and DSC-MRI were performed at baseline and repeated 4–8 weeks post-SRS to predict treatment responses at the midterm follow-up (6–12 months). Midterm outcomes were classified according to RANO-BM criteria as responders (complete response, partial response, or stable disease) or non-responders (progressive disease). Receiver operating characteristic (ROC) analyses evaluated the diagnostic accuracy of individual perfusion parameters and their combinations.

**Results:**

Forty lesions (74%) were responders, while 14 (26%) were non-responders. Progressive lesions showed a mean volume increase of 5.5-fold, whereas responders demonstrated a 60% mean volume reduction. Responders showed significantly lower absolute post-SRS K-trans (median 0.015 vs. 0.035 min⁻¹; *p* = 0.005), a higher proportional decrease in K-trans from baseline (− 27% vs. +13%; *p* = 0.017), and lower post-SRS Ve (*p* = 0.009) compared to non-responders. Absolute post-SRS K-trans had the highest individual predictive accuracy (AUC = 0.75, accuracy = 78%, sensitivity = 86%, specificity = 55%). Neither the dynamic change nor post-SRS nCBV alone predicted midterm response; however, combining post-SRS nCBV with K-trans slightly improved predictive performance (AUC = 0.76, accuracy = 79%).

**Conclusion:**

Early post-SRS absolute K-trans is the best perfusion parameter for predicting midterm response in lung carcinoma BMs. DSC-MRI parameters alone offer limited predictive value.

## Introduction

Brain metastases (BM) of lung carcinoma are the primary leading metastatic brain involvement, accounting for 50% of all brain metastases [1, 2]. The prognosis of patients with BM of lung carcinoma remains poor despite the recent advances in systemic treatment [3]. Stereotactic radiosurgery (SRS) is the preferred modality in non-surgical tumors, securing healthy brain parenchyma, and efficient in achieving local control [4]. Predicting the SRS response remains challenging, especially after six months as viable tumor recurrence is not uncommon [5, 6]. For this reason, imaging biomarkers in addition to the conventional brain MRI can be useful to distinguish midterm responders from nonresponder BM of lung carcinoma.

Dynamic contrast-enhanced MR imaging (DCE-MRI) and dynamic susceptibility contrast MR imaging (DSC-MRI) have been thoroughly investigated to assess tumor microvascularity and its association with treatment outcomes. Parameters such as volume transfer constant (K-trans), extracellular vascular space (V_e_), and plasma volume fraction (V_p_) provide insights into vascular permeability and the presence of angiogenesis within the tumors [7]. DSC-MRI-based normalized cerebral blood volume (nCBV) gives information about the volume of blood passing through the tumor at a certain time, serving as a surrogate marker for tumor-induced angiogenesis [8]. The prior studies have reported the utility of those perfusion imaging techniques in distinguishing radionecrosis from tumor progression, the perfusion imaging markers anticipating the late-phase evolution of irradiated BM are still underexplored.

The study aims to assess the predictive value of early post-SRS DCE- and DSC-MRI parameters along with the effects of dynamic changes in these parameters, to distinguish SRS responders and non-responders at the midterm follow-up. We hypothesize that early alterations in these perfusion metrics may be correlated with midterm tumor local control, thus providing prognostic information to patients with lung carcinoma BM undergoing SRS.

## Material & method

### Patients and treatment method

This study was performed at a tertiary center between March 2023 and July 2024 with a retrospective design. The local clinical ethical committee approved the institutional review board and the requirement for informed consent was waived due to the retrospective nature of the study. The study population consists of participants with BM of lung carcinoma who underwent SRS.

The inclusion criteria of the study were as follows: (1) Participants with at least one cerebral metastasis who had baseline comprehensive brain MRI with contrast, including DCE and DSC perfusion imaging, followed by surveillance control brain MRI using the same perfusion imaging protocol within 4–8 weeks after SRS. (2) participants who had follow-up brain MRI with contrast at least 6 months after the SRS. Exclusion criteria were as follows: (1) patients who have undergone prior irradiation to the targeted lesion before the study period. (2) Recurrent metastasis along the metastasectomy cavity or prior radiotherapy site. (3) Vascular access limitations that impeded contrast administration and compromised the DCE and DSC perfusion imaging in both pre-SRS and post-SRS brain perfusion MRI. Although whole-brain radiotherapy (WBRT) is generally considered the standard of care for brain metastasis of small cell lung carcinoma (SCLC), our institution employs SRS in cases with a limited number of lesions (typically ≤ 5 BMs), controlled extracranial disease, favorable anatomical location, and in most cases patient preference [9]. This practice aims to preserve quality of life by minimizing the neurocognitive side effects commonly associated with WBRT.

The midterm phase was defined as between 6 and 12 months after the SRS to the targeted BM [10, 11]. Based on midterm outcomes assessed at least six months following SRS, the BMs were classified into two groups: responders and non-responders. The responders were defined as the BM showing complete response (CR), partial response (PR), or stable disease (SD) at the midterm stage after the SRS. Based on RANO-BM criteria, the CR was referred to as the complete resolution of BM after the SRS. The PR was characterized by a minimum of 30% reduction in lesion volume at least > 4 weeks after the SRS. Local control or SD, also included in the responder BMs group, was defined as a change of < 30% decrease or < 20% increase of BM volume with reduced surrounding edema compared to the pre-SRS BM volume [12]. This definition of a satisfactory response was further supported by comprehensive clinical evaluations during follow-up visits, ensuring that radiological changes aligned with clinical improvement. The non-responders were defined as having both radiological progression or clinical deterioration at the midterm phase after SRS. Radiologically progressive lesions are defined as an increase in BM volume of more than 20% of pre-treatment volume, accompanied by pronounced surrounding edema or the evolution of new solid components at the midterm phase [12, 13]. Clinical deterioration was characterized by the emergence or worsening of neurological symptoms attributable to the treated brain metastasis—such as new-onset seizures, persistent or worsening headache, cognitive decline, or focal neurological deficits. These focal deficits included motor weakness (e.g., hemiparesis or monoparesis), sensory disturbances, speech or language impairments (aphasia or dysarthria), visual field defects, or gait imbalance. All such symptoms necessitated further neuroimaging at the midterm phase to evaluate for potential treatment failure or progression. To be classified as non-responders in the current study, those patients were also required to meet the criteria for radiological progression in the interim brain MRI as defined by the RANO-BM guidelines.

Lesion volumes were measured at the initial phase before SRS, early post-SRS stage, and midterm phase (≥ 6 months) with the following formula *4/3 x π x d*_*1*_*/2 x d*_*2*_*/2 x d*_*3*_*/2.*

### DSC and DCE MRI acquisition protocols

All examinations were performed using a 1.5T MRI scanner (Aera, Siemens Healthcare, Erlangen, Germany) with a 32-channel phased-array head coil. DCE-MRI and DSC-MRI were performed followed by conventional brain MRI. For this purpose, the DCE-MRI was first administered with 0.1 mmol/kg Gadobutrol at the rate of 5 ml/s followed by 25 ml isotonic saline infusion. DSC-MRI follows this with the same contrast administration strategy.

DCE-MRI acquisition was performed with the axial plane with a spoiled gradient echo T1-weighted sequence by utilizing the following parameters; repetition time (TR)/echo time (TE) 5.08/1.82 ms; field of view (FOV) 220 × 192 mm, slice thickness (ST) 3.5 mm and flip angle (FA) 15º, temporal resolution of 7 s, and overall acquisition time 255 s.

DSC-MRI acquisition was performed with the axial plane using an echo-planar imaging sequence by using the following parameters; TR/TE 2265/30 ms; FOV 224 × 224 mm; ST 3 mm; FA 75º; voxel size 2.30 × 2.40 × 4.00 mm. The best arterial input factor was manually selected from the right middle cerebral artery by choosing the highest amplitude mean-time curve to obtain the most appropriate rCBV mapping.

Axial T1-vibe postcontrast imaging was performed after the completion of DSC-MRI and DCE-MRI. The axial postcontrast T1-vibe was performed with ST of 1.5 mm; matrix size of 270 × 320 mm; FOV, 198 × 230 mm; TR/TE 8/2.5 ms; and FA 10º.

### Image processing and analysis of DCE and DSC-MRI parameters

DSC and DCE-MRI perfusion image raw data were transferred to the Philips IntelliSpace Portal environment to build colorized K-trans, Ve, Vp, and rCBV parameter mapping. The Extended Tofts Linear model was used to generate those permeability parameters [14].

All regions of interest (ROI) were drawn by delineating the enhancing lesion in axial post-contrast T1-vibe, followed by semi-automatically registering the delineated ROI to the derived K-trans, Ve, Vp, and rCBV map. Special attention was given to accurately outlining the optimal ROI around the small cerebral lesion (maximum size < 5 mm) and co-registering the corresponding selected ROI across the above-specified perfusion maps. The lesions’ entire span, including solid portions, was covered by ROI and the exactly similar ROI area was replicated in each perfusion parameter map. The ROI delineation, replication, and transfer processes were performed by a neuroradiologist with 7 years of specialized experience (Y.E.S.). Subsequently, another blinded reviewer, a national board-certified neuroradiologist with 9 years of expertise (A.P.), assessed the replicated ROIs for appropriate size, shape, and conformity to the enhancing lesions in the axial post-contrast T1-vibe images. If any incongruence was encountered, such as mismatching of ROI with the hot-spot part of the lesion or suspect of subtle misposition, in such cases, the subject was not enrolled in the study. The minimum drawn ROI size was 15 mm^2^ for each lesion. Further care was taken not to sample an adjacent vessel, intratumoral hemorrhage, or surrounding edema that could compromise the measurement. The lesion-based rCBV values were transformed to normalized nCBV parameters by dividing the lesion-based rCBV to those of the precisely same copied ROI of contralateral normal-appearing deep white matter.

The DCE and DSC perfusion parameters used in the study provide an insight into the vasculer microenvironment of the metastasis. K-trans (volume transfer constant) quantifies the rate at which contrast moves from plasma into extracellular extravascular space, thus reflecting the permeability and extent of blood-brain barier disruption. Ve (extravascular extracellular space volume fraction) represents the fraction of tissue occupied by extracellular space, which is presumed to give information about tissue cellularity or the proportion of extracellular matrix within the metastasis. Vp (plasma volume fraction) reflects the fractional volume of blood plasma within the metastasis and may serve as a marker of vascular density [15]. The nCBV parameter, derived from DSC perfusion imaging, estimates tumor blood volume by calculating the area under the curve of rapid susceptibility signal changes over time. As such, nCBV (normalized cerebral blood volume) provides indirect information about angiogenesis, reflecting the dynamic passage of contrast media into the tissue and its vascular characteristics [16].

### Radiosurgery protocol

All patients were treated with Leksell Gamma Knife^®^ Icon™ (Elekta Instrument AB, Stockholm, Sweden) or Elekta Versa HD™ (Elekta Instrument AB, Stockholm, Sweden). Each lesion was treated with a tailored SRS protocol based on lesion size, location, and patient comorbidities. Single-fraction SRS was delivered in 38 of 54 lesions (70%) with a median dose of 20 Gy (range: 18–24 Gy). The remaining 16 lesions (30%) received fractionated SRS over 1–10 sessions, with total doses ranging from 20 to 30 Gy (median per-fraction dose: 5 Gy). All patients adhered to the institutional protocol, suggesting a 10-day washout period before and after SRS to suspend or initiate systemic treatment [17]. This approach is intended to temporally separate systemic treatments, such as chemotherapy or targeted therapies, from the SRS course. The rationale behind this protocol is to minimize the risk of overlapping toxicities and to enhance treatment compliance [18].

### Statistical analysis

All statistical analyses were conducted using IBM SPSS Statistics (Armonk, NY, USA). Normality was assessed with the Shapiro-Wilk test. Depending on the data distribution, outcomes for responders and non-responders were compared using either the independent samples Student’s t-test or the Mann-Whitney U test. The Wilcoxon signed-rank test was applied to evaluate changes in nonparametric perfusion parameters before and after SRS. A p-value of < 0.05 was considered statistically significant. Receiver operating characteristic (ROC) curve analysis was performed for perfusion parameters that were statistically significant in univariate analysis. The optimal diagnostic performance for each parameter was identified using Youden’s index. The bivariate logistic regression model was generated to obtain the exponential odds of predicted probabilities from the combination of DCE-MRI and DSC-MRI parameters. ROC analysis was used to assess the performance of those combined tests to the SRS response at the midterm phase. The classification performance metrics, including sensitivity, specificity, and accuracy, were calculated. Additionally, key statistical parameters, such as regression coefficients, odds ratios, and Wald statistics were also derived.

## Results

### Baseline characteristics

The study included 26 (16 F/10 M) participants with 54 eligible BMs of lung carcinoma. The mean age was 62 ± 10 years. Among them, 18 participants with non-small cell lung carcinoma (NSCLC) had 36 BMs, while 8 participants with small cell lung carcinoma (SCLC) had 18 BMs. Table 1 summarizes the study population’s demographics, clinical characteristics, and the SRS response status at the midterm phase.

**Table 1:** Baseline characteristics and the SRS response status

Variable	Outcome
Number of Patients, *n* = 26, %	
*SCLC*	8 (31)
*NSCLC*	18 (69)
The histology of brain metastasis, *n* = 54, %	
*SCLC*	18 (33)
*NSCLC*	36 (66)
Age (y.), mean ± SD,	62 ± 10
Gender (Female / Male), *n* = 26	16 /10
BMs type, *n* = 54, %	
*Solid*	35 (65)
*Cystic*	19 (35)
Midterm (6–12 mo.) SRS outcome of BMs, *n* = 54, %	
*Complete or Partial response*	32 (59)
*Stable disease*	8 (15)
*Progressive metastasis*	14 (26)
State of midterm SRS response, *n* = 54, %	
*Responders*	40 (74)
*Non-responders*	14 (26)

BM, brain metastasis; SCLC, small cell lung carcinoma; NSCLC, non-small cell lung carcinoma; SRS, Sterotactic radiosurgery

### Midterm response status of brain metastasis

**Fig. 1:**
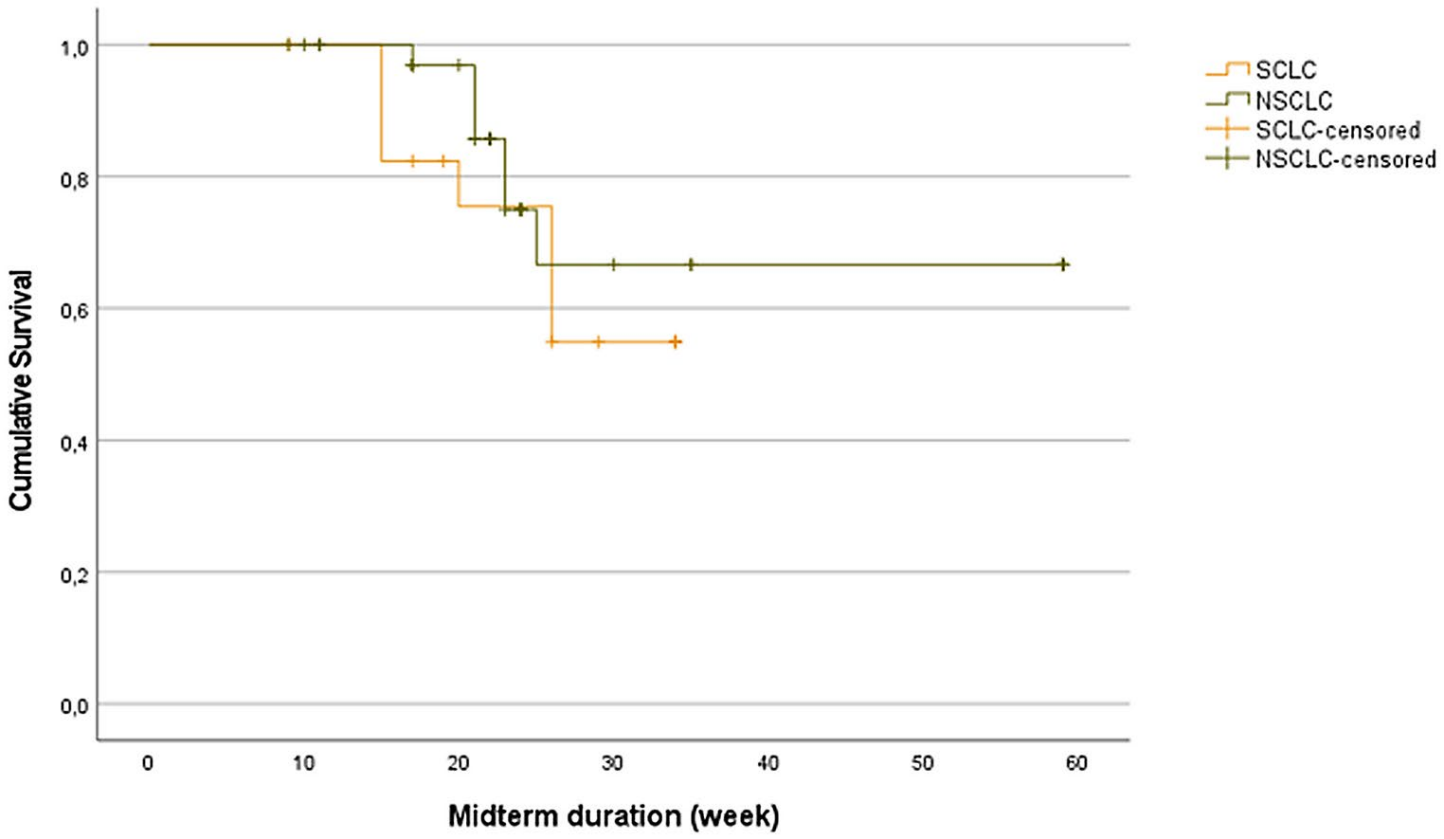
Lesion-based progression-free survival after the SRS. No difference was noted in the response status of the cerebral metastasis based on tumor subtype, (*p* = 0.46). *SCLC*,* Small cell lung carcinoma; NSCLC*,* non-small cell lung carcinoma*

A total of 54 BMs of lung carcinoma from the 26 patients met the study’s eligibility criteria. The average midterm follow-up time was 26 weeks. Forty of the BMs (74%) showed satisfactory responses, including 32 (59%) complete or partial responses and 8 (15%) stable lesions representing local control as determined by RANO-BM criteria. Out of 32 SRS-responsive BMs, 20 showed complete resolution without residual enhancement, while 12 BMs were considered the partial response, among which the majority transformed into cystic cavities with more than 30% reduction in tumor volume at the midterm phase. Conversely, 14 (26%) cases showed radiologically progressive disease at the midterm phase. The lesion-based progression-free survival was not different between the BM of SCLC or NSCLC, as demonstrated in Fig. 1 (*p* = 0.43).

We also assessed whether response status correlated with the administered radiation dose or fractionation scheme. Among the 40 responding lesions, 30 (75%) were treated with single-fraction SRS at doses ≥ 18 Gy, while only 8 (57%) of the 14 non-responding lesions received such doses. Although a trend toward better response with higher single-fraction doses was observed, the association did not reach statistical significance (*p* = 0.19). There was no significant difference in total dose or fraction number between responders and non-responders.

### Perfusion outcomes of the cerebral metastasis before and early after the SRS

**Fig. 2:**
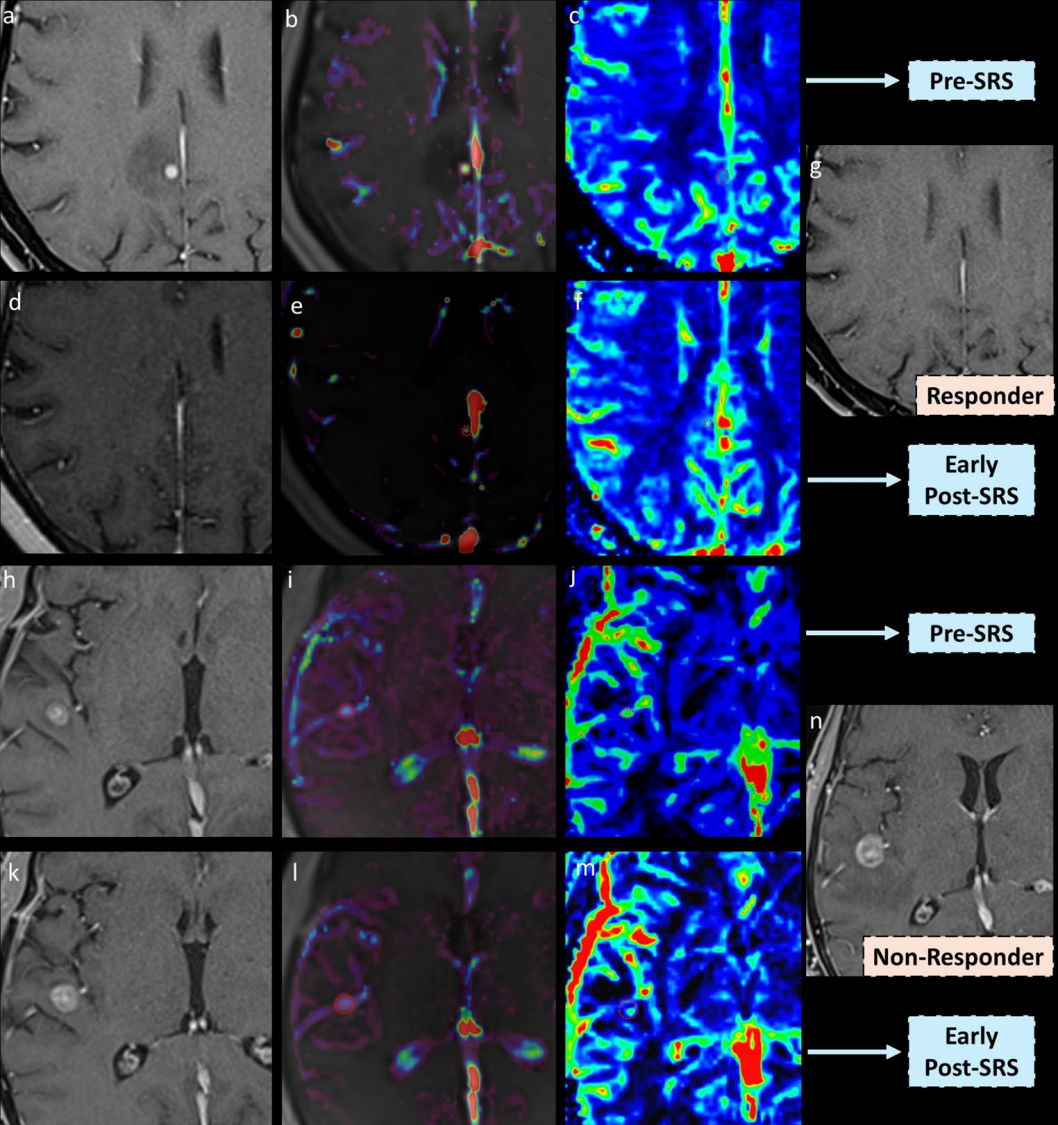
Representative cases of stereotactic radiosurgery (SRS) responder and non-responder brain metastases (BMs) from lung carcinoma, demonstrating multiparametric MRI findings at pre- and early post-SRS timepoints. **(a)** In the responder case, a 66-year-old patient with lung adenocarcinoma had a solid metastasis located in the right deep parietal white matter (precuneus). **(b)** Pre-SRS K-trans was measured at 0.058 min⁻¹, and **(c)** the normalized cerebral blood volume (nCBV) was 3.4 with the same region of interest (ROI). **(d)** Four weeks after SRS, there was a marked reduction in lesion size and perilesional edema. **(e)** The early post-SRS K-trans decreased to 0.042 min⁻¹, corresponding to a 28% reduction. **(f)** nCBV of BMs decreased to 2.9 at the early post-SRS stage. **(g)** At the 28-week midterm follow-up, the lesion had completely resolved with regressed surrounding edema consistent with a complete treatment response. **(h)** In the non-responder case, a 55-year-old patient with small cell lung carcinoma had a solid metastasis in the right superior temporal gyrus. **(i)** Pre-SRS K-trans of the BMs was 0.121 min⁻¹, and **(j)** the corresponding nCBV was 3.9 with the same ROI. **(k)** Early post-SRS imaging showed a slight increase in lesion size, **(l)** with a persistently high K-trans of 0.125 min⁻¹, and **(m)** a modest reduction in nCBV to 3.2. **(n)** At the 24-week midterm follow-up, the lesion is enlarged further with prominent surrounding edema, necessitating an additional SRS session. In this non-responder case, persistently high K-trans despite a mild nCBV decrease suggested treatment resistance and poor response to SRS

**Fig. 3:**
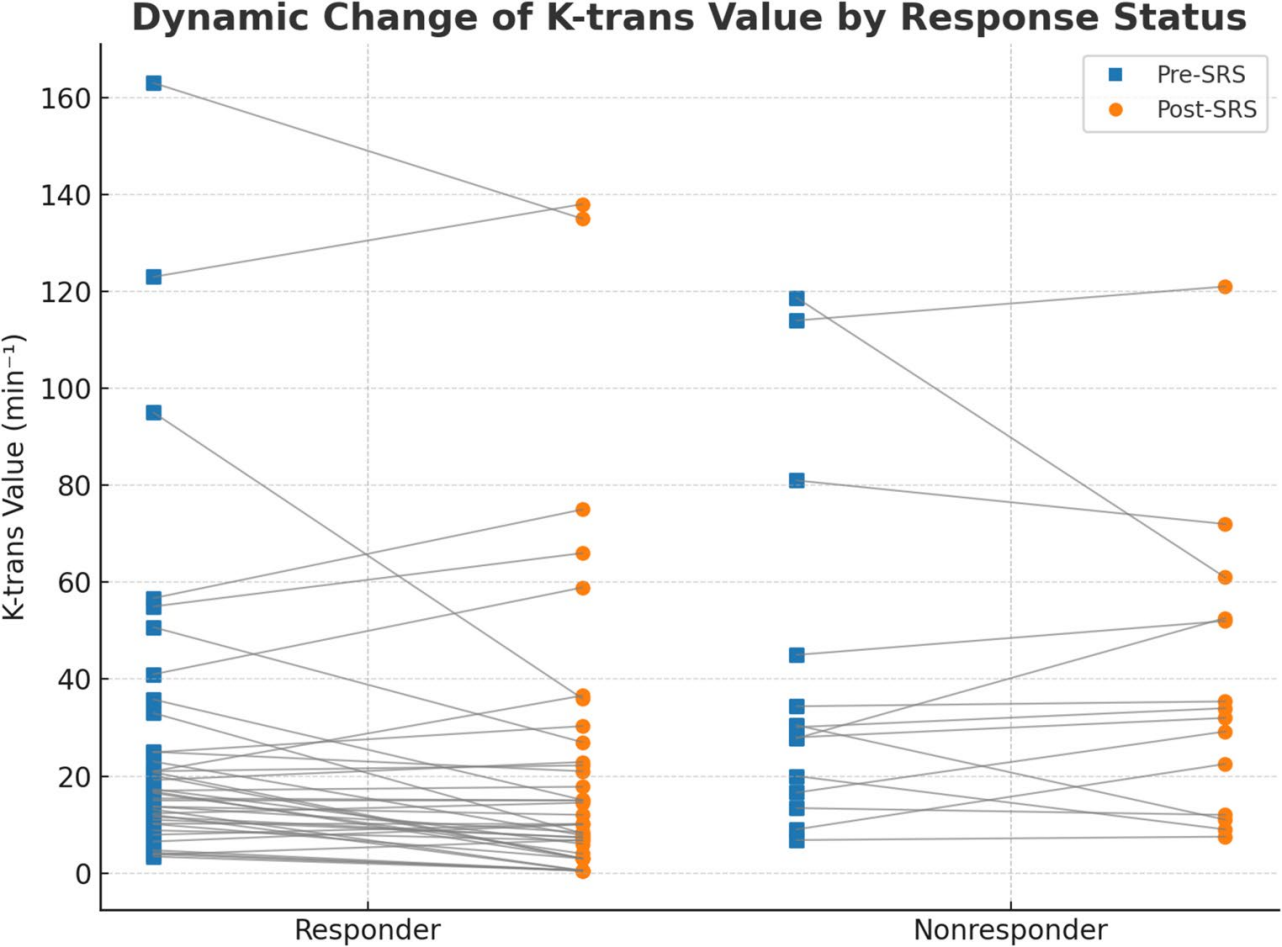
Lesion-level illustration of dynamic changes in K-trans values following stereotactic radiosurgery (SRS) for brain metastases (BMs) from lung carcinoma. Each line represents an individual BM, depicting the K-trans change from pre-SRS to early post-SRS evaluation. The responder group (*n* = 40) demonstrated a mean K-trans reduction of 27% ± 50%, whereas the non-responder (*n* = 14) group showed a mean increase of 13% ± 52% (*p* = 0.017)

**Fig. 4:**
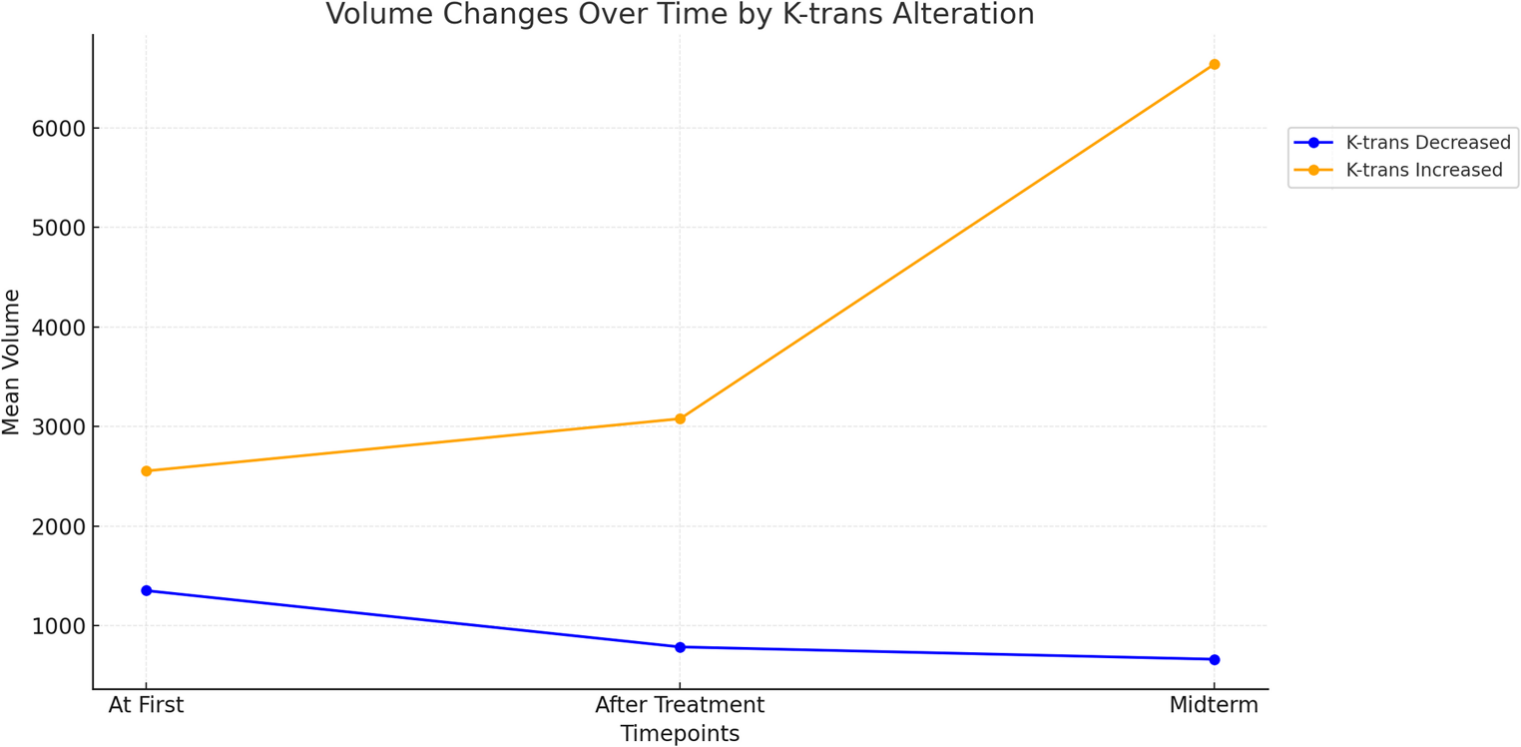
Volumetric changes in lung carcinoma cerebral metastases stratified by K-trans alterations 4–8 weeks after SRS. Tumors with increased K-trans showed significantly higher volumes at the midterm time point compared to those with decreased K-trans (*p* = 0.004)

Representative DCE-MRI and DSC-MRI outcomes of the BM are listed in Table 2. An example of responder and non-responder BMs at the midterm stage is illustrated in Fig. 2. The median absolute Post-SRS K-trans was 0.015 in the responder and 0.035 in the nonresponder group (*p* = 0.005), while there was no difference in Pre-SRS K-trans among the groups (*p* = 0.058). Twenty-seven (67.5%) of the responder BMs showed a reduction of the K-trans, while 9 (64.3%) of the non-responder BMs had increased K-trans after the SRS. The proportion of the K-trans change after the SRS was − 27% ± 50 in the responder BMs and 13% ± 52 in the nonresponder BMs, (*p* = 0.017). Figure 3 shows the lesion basis change of K-trans value before and early after (4–8 weeks) the SRS both in responder and nonresponder group. The median Pre-SRS V_e_ and Post-SRS V_e_ significantly differ between the responder and non-responder BMs, as outlined in Table 2. However, unlike the absolute ratio of K-trans change, the proportion of V_e_ change was not different between the responder and non-responder BMs (*p* = 0.15). The same ROI-based pre-SRS and post-SRS nCBV of the responder BMs were not significantly different from those of the non-responder BMs. Tumor volume change at the midterm phase was associated only with K-trans alteration among all DCE-MRI and DSC-MRI parameters as demonstrated in Fig. 4 (*p* = 0.004). Post-SRS nCBV or the other DCE-MRI parameters had no association with midterm tumor volume change.

**Table 2:** Stratification of DCE and DSC parameters in brain metastases before and early after SRS according to midterm treatment response

	Responder BMs, *n* = 40	Non-responder BMs, *n* = 14	*p*-value
Pre-SRS K-trans, median [IQR]	0.019 [0.025]	0.030 [0.064]	0.058
Post-SRS K-trans, median [IQR]	0.015 [0.026]	0.035 [0.031]	0.005
K-trans change, (%), mean ± SD	-27 ± 50	13 ± 52	0.017
Pre-SRS V_e,_ median [IQR]	0.078 [0.175]	0.172 [0.200]	0.024
Post-SRS V_e,_ median [IQR]	0.120 [0.245]	0.209 [0.134]	0.009
V_e_ change (%), median [IQR]	-17 [130]	24 [48]	0.15
Pre-SRS V_p,_ median [IQR]	3.3 [3.8]	4,6 [2.6]	0.28
Post-SRS V_p,_ median [IQR]	3.8 [3.5]	4.2 [1.4]	0.23
V_p_ change (%), median [IQR]	-4.8 [137]	6.2 [67]	0.98
Pre-SRS nCBV, median [IQR]	1.5 [2.0]	2.0 [2.3]	0.26
Post-SRS nCBV, median [IQR]	1.0 [1.2]	1.7 [2.3]	0.17
nCBV change (%)	-30 [141]	17 [210]	0.59

BMs, Brain metastasis; SRS, Stereotactic radiosurgery; K-trans, time-dependent leakage; K_ep_, reflux rate constant; V_e_ fractional extravascular extracellular space volume; V_p,_ fractional plasma volume; nCBV, normalized cerebral blood volume

### Predictive value of perfusion parameters for midterm response to SRS

**Fig. 5:**
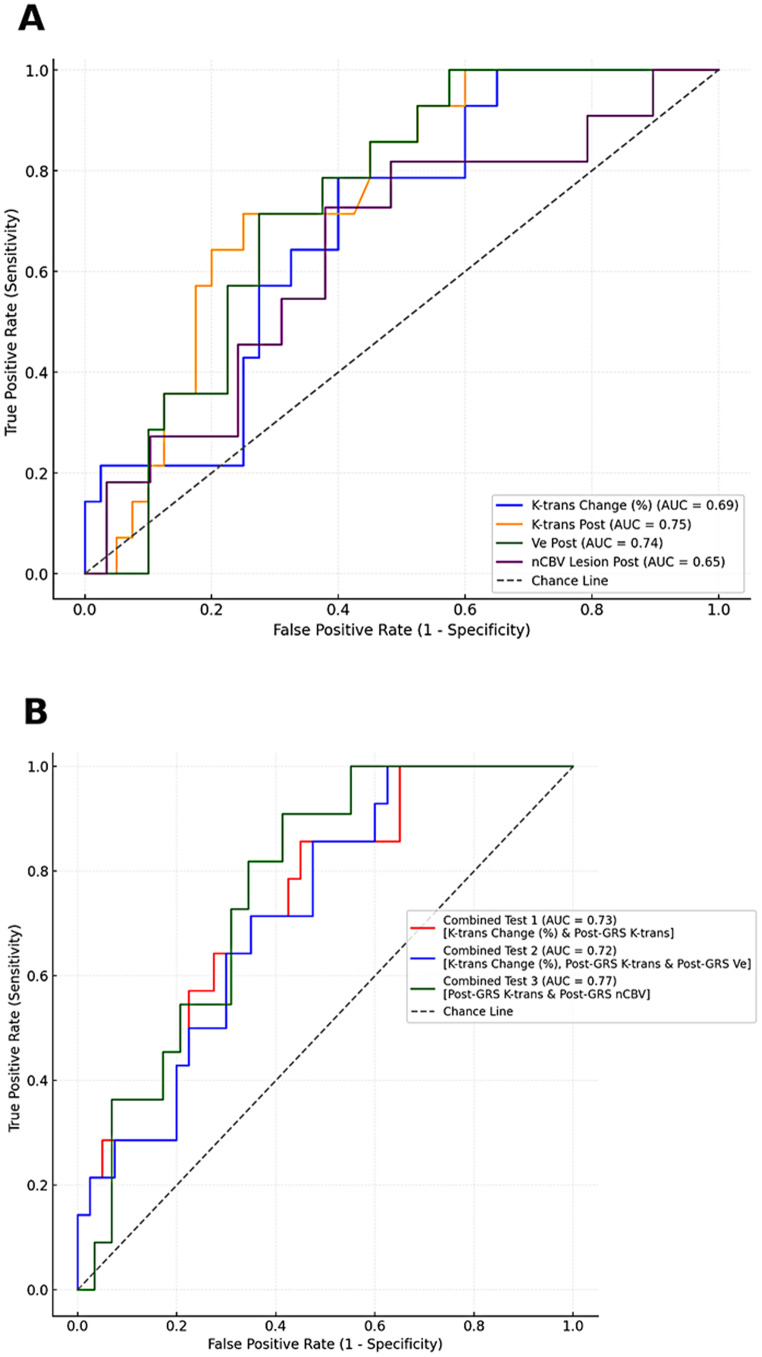
Receiver operating characteristics (ROC) analysis for the significant parameters at 4–8 weeks after the SRS in terms of midterm response of lung carcinoma cerebral metastasis. **(A)** ROC curves of absolute post-SRS K-trans, V_e,_ nCBV value, and proportion of K-trans change from the pretreatment stage. **(B)** ROC analysis of combined tests between the significant DCE-MRI and DSC-MRI parameters

The parameters identified as statistically significant in the univariate analysis were further evaluated using ROC analysis. Representative ROC curves for the significant DCE-MRI and DSC-MRI parameters are presented in Fig. 5a.

The absolute post-SRS K-trans demonstrated good predictive performance, with an AUC of 0.75 and an accuracy of 78%. A cut-off value of 0.011 for post-SRS K-trans achieved a sensitivity of 86% and specificity of 55%, corresponding to a Youden index of 0.413. In contrast, the proportional change in K-trans was less effective in predicting the SRS response (AUC = 0.70, accuracy = 74%). The optimal cut-off for midterm SRS response prediction was a reduction in K-trans of more than 10%, yielding a sensitivity of 79% and specificity of 60% (Youden index = 0.390).

The absolute post-SRS Ve showed nearly similar sensitivity (79%) and specificity (60%) to the proportional K-trans change, yet was less effective than the absolute post-SRS K-trans (AUC = 0.73, accuracy = 72%) at a cut-off of 0.090. Neither the nCBV change nor the absolute post-SRS nCBV alone effectively predicted the midterm SRS response of BMs with lung carcinoma. However, combining post-SRS nCBV with post-SRS K-trans slightly improved the diagnostic performance, achieving the highest accuracy compared to other parameter combinations. Incorporating the adjunctive nCBV with the best cut-off of 1.3 enhanced the predictive value of post-SRS K-trans (cut-off: 0.011 min^− 1^) for distinguishing the midterm SRS response from tumor progression (AUC = 0.77, accuracy = 79%). Table 3 summarizes the performance of various combinations of the significant DCE and DSC-MRI parameters with the corresponding ROC curves illustrated in Fig. 5b.

**Table 3:** Combined test performance results

Test	AUC	Accuracy	Best Threshold	Sensitivity	Specificity	Wald Statistic	Coefficient	Odds Ratio	*P*-value
Combined test 1	0.73	0.78	0.22	0.86	0.59	5.44	4.86	128.58	0.02
Combined test 2	0.72	0.78	0.21	0.86	0.53	5.49	4.86	129.32	0.019
Combined test 3	0.76	0.79	0.25	0.82	0.66	2.43	4.3	73.37	0.045

*Combined test 1;* K-trans change and Post-SRS K-trans

*Combined test 2*; K-trans change, Post-SRS K-trans, and Post-SRS V_e_

*Combined test 3;* Post-SRS K-trans and Post-SRS nCBV

*AUC;* Area under the curve

## Discussion

This study assessed the utility of early DCE-MRI and DSC-MRI, performed between 4 and 8 weeks after SRS, in predicting midterm response status at least six months later. Despite the longer acquisition time and challenges in maintaining consistency, our preliminary findings indicate that early post-SRS DCE-MRI, specifically the K-trans parameter for lung carcinoma BMs, demonstrates better predictive capability than the nCBV for midterm SRS response assessment. A post-SRS K-trans threshold of 0.011 min⁻¹ achieved a high sensitivity of 88%, albeit with limited specificity. The combination of nCBV with post-SRS K-trans of BMs slightly improved the specificity of predicting midterm SRS response. These initial findings might suggest that even a modest increase in the K-trans early after the SRS might be correlated with treatment resistance or midterm tumor progression.

In our study, we observed a reduction in K-trans following SRS in the responder group, whereas the resistant group showed no significant change; instead, a slight increase in K-trans was noted within 4–8 weeks post-SRS. Another study involving various BMs identified a cut-off of 15% increase in K-trans after radiosurgery, yielding a sensitivity of 78% and specificity of 85%. The same cohort reported that a 1% increase in K-trans was associated with a 50% relative risk of midterm tumor progression [10]. However, unlike that study, our findings suggest that neither K-trans nor other DSC- or DCE-based perfusion parameters were highly specific for predicting later tumor progression. Notably, our cohort consists exclusively of SCLC and NSCLC cases and has a longer mean imaging surveillance period (> 24 weeks). Another cohort with 53 BM of lung carcinoma reported that an absolute post-radiotherapy K-trans threshold of 0.017 min⁻¹ could distinguish responders from non-responders with 89% sensitivity and 67% specificity, whereas the relative change in K-trans was not associated with midterm tumor response [17]. Our study identified a lower K-trans threshold of 0.011 min⁻¹, demonstrating high sensitivity but limited specificity. However, we found that the dynamic changes in K-trans following SRS were valuable in predicting the potential SRS resistance.

Our findings emphasize the limited predictive value of nCBV alone in anticipation of midterm response following SRS of lung carcinoma with BMs. However, integrating an nCBV threshold of 1.3 with post-SRS absolute K-trans within 4–8 weeks slightly enhanced test performance, increasing specificity from 55 to 66% in predicting midterm response. A large meta-analysis reported a high pooled performance of nCBV, achieving a sensitivity of 83% and specificity of 78% in differentiating progression from radionecrosis [19]. Notably, the studies included in the meta-analysis exhibited significant variability in the timing of the first post-SRS DSC-MRI. Among them, Mitsuyu et al. and Ju Koh et al. performed DSC-MRI screening 1–3 months after radiosurgery, similar to our protocol, and reported high accuracy rates of 93% and 95%, respectively, indicating near-perfect discriminative power of nCBV [20, 21]. A key difference is that those studies employed the hot-spot technique in irradiated cerebral metastases, whereas our study utilizes a whole-lesion ROI approach. This method minimizes sampling bias when comparing DCE-MRI and DSC-MRI performance, making it more clinically applicable, though it may incorporate cystic-necrotic tissue that evolves post-SRS.

K-trans is a coefficient of contrast leakage into the tissue in the extended Tofts pharmacodynamic model [22]. K-trans can be affected by the local factors in the brain parenchyma such as proportional blood volume, the extent of disruption in the blood-brain barrier, endothelial permeability, and neoangiogenesis in BM [23]. The presumed increase or insufficient reduction in the K-trans is mostly attributable to the persistent neoangiogenesis in the resistant BM, whereby it is likely that the contrast influx rate into the BM is more pronounced than the radionecrosis. Acquisition difficulties and MRI protocol-related variability impair the identification of the absolute cut-off for distinguishing treatment-related changes from the residual tumors, making the DCE-MRI less preferable. However, our current cohort suggests that even slight increases early after the SRS, K-trans can be associated with tumor volume rises within 6–12 months while the dynamic change in nCBV of the DSC-MRI failed to predict midterm tumor volume.

The impact of early radiation-induced tumor apoptosis is most pronounced within the first week, effectively suppressing angiogenic signaling and leading to a rapid decline in vascular permeability post-radiotherapy [24, 25]. This process ultimately results in stable or decreased K-trans and nCBV values. Jabukovic et al. reported that a reduction in K-trans at one week exhibited an 81% specificity in predicting treatment response; however, in contrast to our findings, their series did not reveal an association between K-trans at one month and treatment outcome [26]. Consistent with our results, they also found that nCBV at one month had no predictive value. A key distinction of our cohort is that all participants underwent single-fraction SRS exclusively for cerebral metastases from lung carcinoma, which may explain the discrepancy. The inhibition of angiogenesis could persist beyond one month following SRS, given the high cytotoxic potency of stereotactic radiosurgery modalities such as Gamma knife radiosurgery [27]. This prolonged suppression may contribute to the continuing inhibition of angiogenesis alongside tumor cell death, which can explain the lower K-trans absolute value. This potentially provides a more accurate reflection of midterm tumor response to SRS than one week of immediate DCE-MRI parameters, as adequate time is allowed for targeted metastases to either develop tumor-induced neoangiogenesis or transform into predominant necrosis.

The early post-SRS K-trans value may serve as a practical biomarker to personalize imaging surveillance intervals and inform therapeutic decisions in patients with lung carcinoma brain metastases. Persistently elevated or increasing K-trans levels within weeks after SRS may indicate a higher risk of treatment resistance, warranting closer follow-up or earlier consideration of salvage interventions. In contrast, a significant reduction in K-trans could suggest favorable local control, potentially justifying a less intensive imaging schedule. Although this strategy is not yet supported by sufficient evidence to replace standard post-SRS follow-up protocols, it highlights the potential for individualized radiologic surveillance. In such cases, a DCE-MRI-derived K-trans threshold (e.g., < 0.011 min⁻¹) may represent an actionable marker to guide clinical decision-making.

There were several limitations in our study. First, it was single-centered and retrospective in design. Second, although we performed midterm follow-up assessments between 6 and 12 months, this time frame may not be sufficient to distinguish tumor progression from radionecrosis. Some BMs classified as progressive in the midterm phase may indeed represent transient inflammatory responses or evolving radionecrosis that could have resolved with longer follow-up. While the RANO-BM criteria and clinical evaluation were used to enhance diagnostic accuracy, the absence of extended imaging surveillance remains a limitation. Third, the midterm evaluations were based on lesion evolution and conventional MRI findings, thus, the lack of mid-phase histopathological confirmation of BM is another limitation. Small lesions measuring less than 5 mm were also involved in the analysis, which potentially brings difficulties in lesion-based ROI measurement. Nevertheless, all patients underwent standardized surveillance protocol including 4–8 weeks after the SRS. Moreover, our study compared the dynamic change of both perfusion techniques between the pre-SRS and early post-SRS assessment, seeking the association of permeability alterations to late-phase tumor status rather than providing only absolute values of post-treatment perfusion parameters. Another limitation is the presence of concomitant systemic treatment, which may have influenced the DCE or DSC perfusion parameter or the biological response of brain metastases to SRS. While we cannot eliminate the potential confounding effect of pharmacologic therapies, all patients were managed under a standardized institutional protocol that mandates a minimum 10-day washout period before and after SRS to minimize the acute impact of systemic agents on perfusion imaging. Lastly, despite our primary focus on perfusion biomarkers, we also explored the role of dose and fractionation in treatment response. A higher proportion of responders had received single-fraction SRS with doses ≥ 18 Gy. While this trend aligns with prior evidence supporting higher ablative doses for optimal control, our findings did not reach statistical significance, likely due to sample size limitations. Yet, dose stratification may serve as a confounding variable and should be further examined in future prospective studies. Lastly, while post-SRS K-trans showed the highest individual predictive performance among other perfusion parameters, it did not uniformly decrease among all responder lesions. This highlights the limited capacity of K-trans as a standalone marker and reflects the biological complexity of treatment response. Factors such as transient vascular effects, heterogeneous tumor microenvironments, or delayed therapeutic responses may lead to discordant perfusion trends. Therefore, K-trans should be interpreted as a supportive biomarker in conjunction with clinical and conventional radiological assessment rather than a sole determinant of SRS response.

In conclusion, this study highlighted the moderately favorable predictive performance of K-trans in DCE-MRI for assessing SRS response in brain metastases of lung carcinoma. While proportional changes in K-trans were less effective than post-SRS absolute K-trans, a positive or negative shift in K-trans strongly correlated with tumor volume increase or decrease, respectively, after six months post-SRS. The nCBV change alone did not show an association with midterm response status but slightly improved specificity when combined with post-SRS K-trans assessment. Therefore, only DCE-MRI performed before and 4–8 weeks after SRS may be sufficient to anticipate the SRS response between 6 and 12 months in BMs of the lung carcinoma.

## Data Availability

Data will be provided upon reasonable request.
